# Gain of *ALK* Gene Copy Number May Predict Lack of Benefit from Anti-EGFR Treatment in Patients with Advanced Colorectal Cancer and *RAS-RAF-PI3KCA* Wild-Type Status

**DOI:** 10.1371/journal.pone.0092147

**Published:** 2014-04-01

**Authors:** Filippo Pietrantonio, Claudia Maggi, Maria Di Bartolomeo, Maria Grazia Facciorusso, Federica Perrone, Adele Testi, Roberto Iacovelli, Rosalba Miceli, Ilaria Bossi, Giorgia Leone, Massimo Milione, Giuseppe Pelosi, Filippo de Braud

**Affiliations:** 1 Medical Oncology Department, Fondazione IRCCS Istituto Nazionale dei Tumori, Milan, Italy; 2 Pathology Department, Fondazione IRCCS Istituto Nazionale dei Tumori, Milan, Italy; 3 Unit of Medical Statistics Biometry& Bioinformatics, Fondazione IRCCS Istituto Nazionale dei Tumori, Milan, Italy; University General Hospital of Heraklion and Laboratory of Tumor Cell Biology, School of Medicine, University of Crete, Greece

## Abstract

**Introduction:**

Although *cetuximab and panitumumab show an increased* efficacy for patients with KRAS-NRAS-BRAF and PI3KCA wild-type metastatic colorectal cancer, primary resistance occurs in a relevant subset of molecularly enriched populations.

**Patients and Methods:**

We evaluated the outcome of 68 patients with advanced colorectal cancer and RAS, BRAF and PI3KCA status according to ALK gene status (disomic vs. gain of ALK gene copy number – defined as mean of 3 to 5 fusion signals in ≥10% of cells). All consecutive patients received cetuximab and irinotecan or panitumumab alone for chemorefractory disease.

**Results:**

No ALK translocations or amplifications were detected. ALK gene copy number gain was found in 25 (37%) tumors. Response rate was significantly higher in patients with disomic ALK as compared to those with gain of gene copy number (70% vs. 32%; p = 0.0048). Similarly, progression-free survival was significantly different when comparing the two groups (6.7 vs. 5.3 months; p = 0.045). A trend was observed also for overall survival (18.5 vs. 15.6 months; p = 0.885).

**Conclusion:**

Gain of ALK gene copy number might represent a negative prognostic factor in mCRC and may have a role in resistance to anti-EGFR therapy.

## Introduction

Treatment with anti-epidermal growth factor receptor (EGFR) monoclonal antibodies - cetuximab and panitumumab - improved the outcome of patients with advanced KRAS wild-type colorectal cancer (CRC) in combination with first- or second-line fluoropyrimidine-based chemotherapy or in the setting of chemorefractory disease [1]–[6].

Several resistance biomarkers beyond *KRAS* were studied in order to improve patients selection. It was previously shown that the response rate to cetuximab reached the value of 41.2% for patients with *KRAS*, *BRAF*, *NRAS* and exon 20 *PI3KCA* “quadruple wild-type” status [7]. However, even in molecularly enriched populations, there is still a relevant subset of non responders [8]. The identification of additional resistance biomarkers is an unmet clinical need for anti-EGFR treatment personalization in this setting.

Anaplastic lymphoma kinase (ALK) is a member of the insulin receptor family with tyrosine kinase activity, which can activate signal transduction by ligand binding, gene amplification or mutation [9]. The discovery of a new potentially relevant oncogenic event in lung cancer, the *EML4-ALK* translocation, and the development of ALK inhibitors with promising results in preclinical models and randomized clinical trials provides the rationale for the comprehensive characterization of *ALK* abnormalities in patients with other solid tumors, such as CRC [10], [11]. Alterations of ALK may interfere with the biological activity of EGFR through cross-talk of signaling pathways. In fact, oncogenic *ALK* may activate independently downstream pathways including the *PI3KCA*/Akt and *RAS-RAF-MAPK*, even in presence of EGFR blockage [12].

The aim of our analysis was to evaluate the role of gain of *ALK* gene copy number in terms of the response rate, progression-free survival (PFS) and overall survival (OS) in patients treated with irinotecan and cetuximab or panitumumab monotherapy for advanced, chemorefractory CRC and wild-type *RAS-RAF-PI3KCA* status.

## Patients and Methods

### Patient population

Sixty-eight consecutive patients with histologically proven metastatic CRC with *KRAS*, *NRAS*, *BRAF*, *PI3KCA* wild-type status were prospectively collected from 2007 to 2013 at “Fondazione IRCCS Istituto Nazionale dei Tumori” and were considered eligible for the present study. Patients received a combination of cetuximab and irinotecan or panitumumab after clinical evidence of refractoriness to standard chemotherapy including fluoropyrimidines, oxaliplatin and irinotecan. The Institutional Review Board of “Fondazione IRCCS Istituto Nazionale dei Tumori” approved this study and all subject signed written informed consent.

### Mutational analysis of RAS-RAF-PI3KCA

Formalin-fixed paraffin-embedded tumour tissues were reviewed for quality and tumour content. A tissue containing at least 80% of neoplastic cells was selected for each case. Macrodissection of 7 μm methylene blue-stained sections allowed the separation of neoplastic and normal cells. Genomic DNA was extracted using the Qiamp FFPE DNA kit (Qiagen, Chatsworth, CA, USA) following the manufacturer's instructions. Mutational analysis of *KRAS* exons 2, 3 and 4 was performed as previously described [13], [14]. *KRAS* exon 2 status was further confirmed through a specific mutant enriched polymerase chain reaction (PCR), known to be a more sensitive approach [15]. *BRAF* (exon 15), *NRAS* (exons 2 and 3) and *PI3KCA* (exons 9 and 20) mutational analysis was performed by means of PCR using specific primers previously described [13], [15]. The PCR products were subjected to direct sequencing using an ABI Prism 3500 DX Genetic Analyzer (Applied Biosystems, Foster City, CA, USA) and then evaluated by means of the ChromasPro software.

### ALK gene copy number status

Three to four μm-thick sections were cut from paraffin blocks and mounted on positively charged slides and dried at least 1 hour at 56°C. Tissue sections were deparaffinized in xylene (3 times each of 10 minutes), rehydrated with an ethanol-to-water series (100%–85%–70%). Subsequently, the sections were pretreated in TE (Tris 5 mM-EDTA 1 mM, pH = 7) at 96° for 15 minutes, rinsed in distilled water and enzymatically digested with pepsin 0,4% in 0.01 N HCl for 6 to 10 minutes at 37°C, with monitoring of the progression of the enzymatic digestion using a phase contrast microscope.

Slides were then washed in distilled water for two times each of 5 minutes, dehydrated in 96% ethanol for 3 minutes, air dried. After application of the probe (ALK FISH DNA Probe, Split signal Dako) on the area of interest the specimens were codenatured at 85°C for 1 minute and then hybridized at 37°C overnight using a Hybridizer (Dako). The following day, coverslips were removed and slides were immersed in posthybridization solution 2XSSC/0.3% NP40 (73°C for 2 minutes) subsequently in 2XSSC/0.1% NP40 (1 minute at room temperature) and finally brifly rinsed in distilled water. The slides were then left to dry in the dark at room temperature, and nuclei were counterstained in Vectashild Antifade solution with DAPI (4,6-diamino-2-phenyindole-2-hydrocloride) (Vector Laboratories, Inc. Burlingame CA).

A minimum of 60 non-overlapping nuclei of invasive tumor cells were scored using Olympus epifluorescence microscope equipped with an 100× oil immersion objective and 4,6-diamidino-2-phenylindole/Spectrum Green/Orange single and triple bandpass filters. The two DNA probe within ALK FISH DNA Probe, Split Signal, are designed to hybridize upstream and downstream of the breakpoint cluster region. Co-localization of the probes results in a yellow signal, whereas translocation events in the breakpoint cluster region will split one signal in separate green (fluorescin) and red (Texas Red) signals. The criteria for ALK translocation positivity was the presence of the split of the probes in at lest 15% of cells.

**Figure 1:**
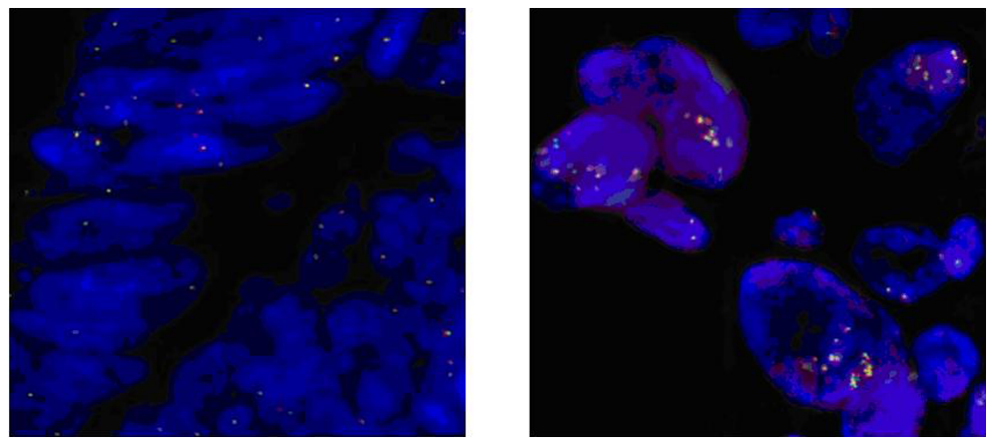
ALK FISH examples. Gain of ALK GCN (including both low and high genomic gain) was defined as a mean of 3 to 5 fusion signals in ≥10% of cells (Figure on right). Disomic cells are shown in Figure on left.

As criteria for copy number aberrations of *ALK* has not been established, we arbitrarily used the following cut-offs adapted from the criteria established for *EGFR* and *HER-2* in non-small cell lung cancer specimens [16], [17]. According to Cappuzzo et al. [16], patients may be classified into six FISH strata with ascending number of copies of the EGFR gene per cell according to the frequency of tumor cells with specific number of copies of the EGFR gene. In our study, we adopted cut-offs for classifying ALK gene copy number alterations as previously described for non-small cell lung cancer [18]. Briefly, gain of *ALK* gene copy number (including both low and high genomic gain) was defined as a mean copy number of 3 to 5 fusion signals in ≥10% of cells and amplification as the presence of ≥6 copies of *ALK* per cell in ≥10% of analyzed cells. In cases where clusters were observed, we reported the percentage of cells with clusters and considered amplified cases with ≥10% of *ALK* clusters [18] (Figure 1).

### Statistical Analysis

Descriptive statistical methodology was used to analyze the results and qualitative data were compared by chi-square test, as appropriate.

PFS was defined as the time from date of enrolment to the date of the first documented progressive disease (PD) or death for any cause. OS was calculated from date of enrolment to the date of death due to any cause, or censored at the date of last follow-up for living patients. Survival curves were plotted by the Kaplan-Meier method and compared by log-rank test. For ordinal variables, a log-rank test of trend was applied. Data analysed were using SPSS version 16.0 for Windows (SPSS, Chicago, IL, USA). Results were reported with 95% confidence intervals (CI). All the statistical tests were conducted at the two-sided 0.05 level of significance.

## Results

### Patient population

**Figure 2:**
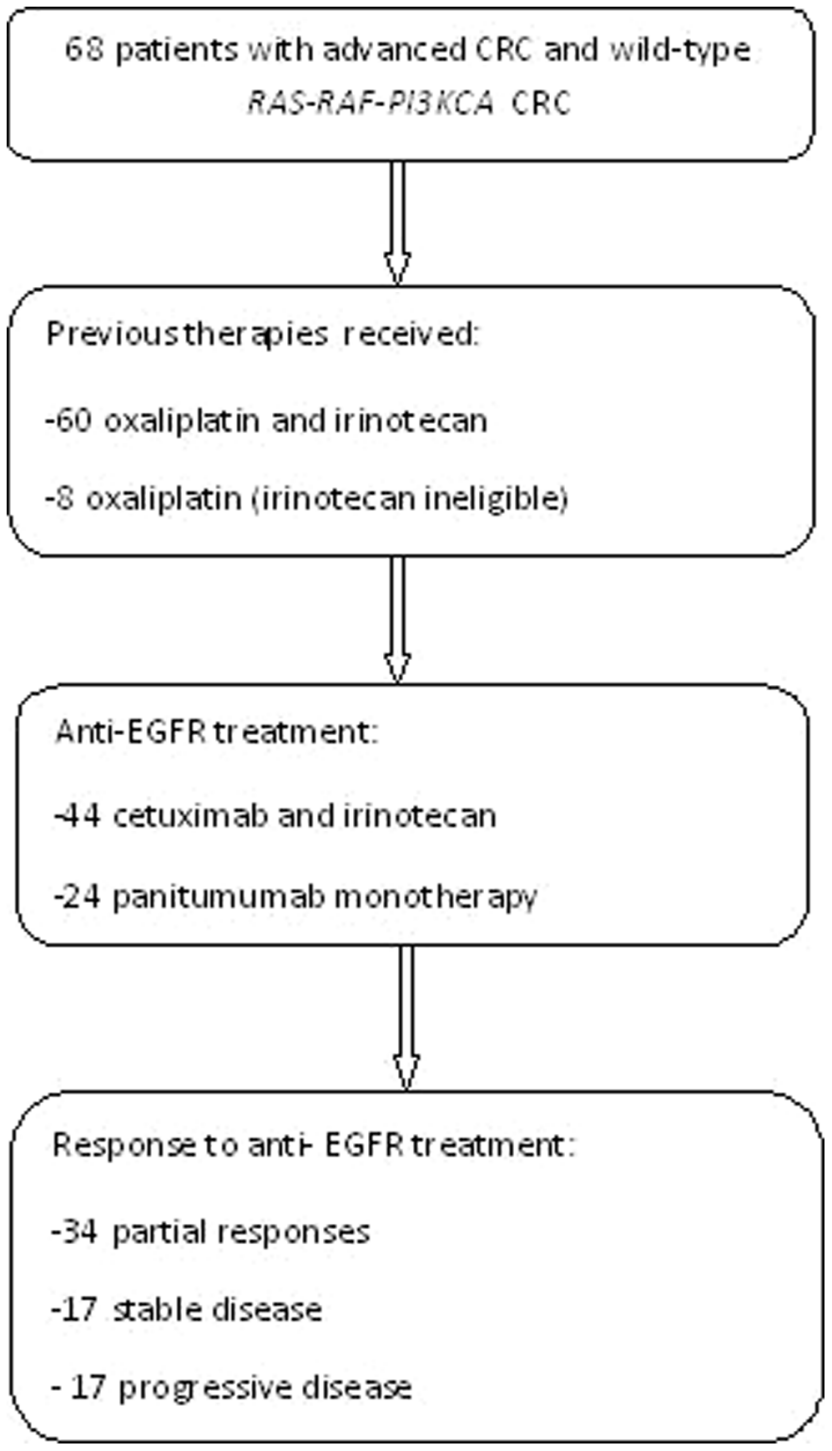
Flow-chart of patients population.

Sixty-eight consecutive patients with advanced wild-type *RAS-RAF-PI3KCA* CRC were included in this prospective dataset at Fondazione IRCCS Istituto Nazionale dei Tumori of Milan. Patient and disease characteristics are shown in Table 1 and summarized in Figure 2. Most of the patients (n = 60, 88%) were classified as chemorefractory and were previously treated with both oxaliplatin-based and irinotecan-based chemotherapy, and after failure of at least two previous lines of chemotherapy. However, 8 (12%) of patients were considered irinotecan ineligible and received panitumumab as second-line treatment after failure of oxaliplatin-based chemotherapy. Overall, we observed a partial response in 34 patients (50%), and progressive disease in 17 cases (25%). Additional 17 patients (25%) showed stable disease (SD), whereas no complete remissions were obtained. Median follow-up of the whole series was 32.5 months. Overall, 67 patients had a documented PD, and a total of 41 (60%) patients died. All deaths were due to PD, while one patient was lost to follow-up. Median PFS and OS were 6.3 and 16.4 months, respectively. The OS curves were truncated at 3 years, namely at a time interval slightly longer than the median follow-up.

**Table 1:** Patients demographics and disease characteristics.

Main characteristics	Number (%)
Age, median (range)	65 (36–81) years
Gender	
Male	38 (56)
Female	30 (44)
ECOG performance status	
0	44 (65)
1	24 (35)
Primary tumour site	
Right colon	15 (22)
Left colon	24 (35)
Rectum	29 (43)
Stage IV presentation	
Synchronous	45 (66)
Metachronous	23 (34)
Sites of disease	
1	22 (32)
≥2	46 (68)
Treatment	
Irinotecan - cetuximab	44 (65)
Panitumumab	24 (35)

### Patients outcome according to ALK gene copy number status

**Figure 3:**
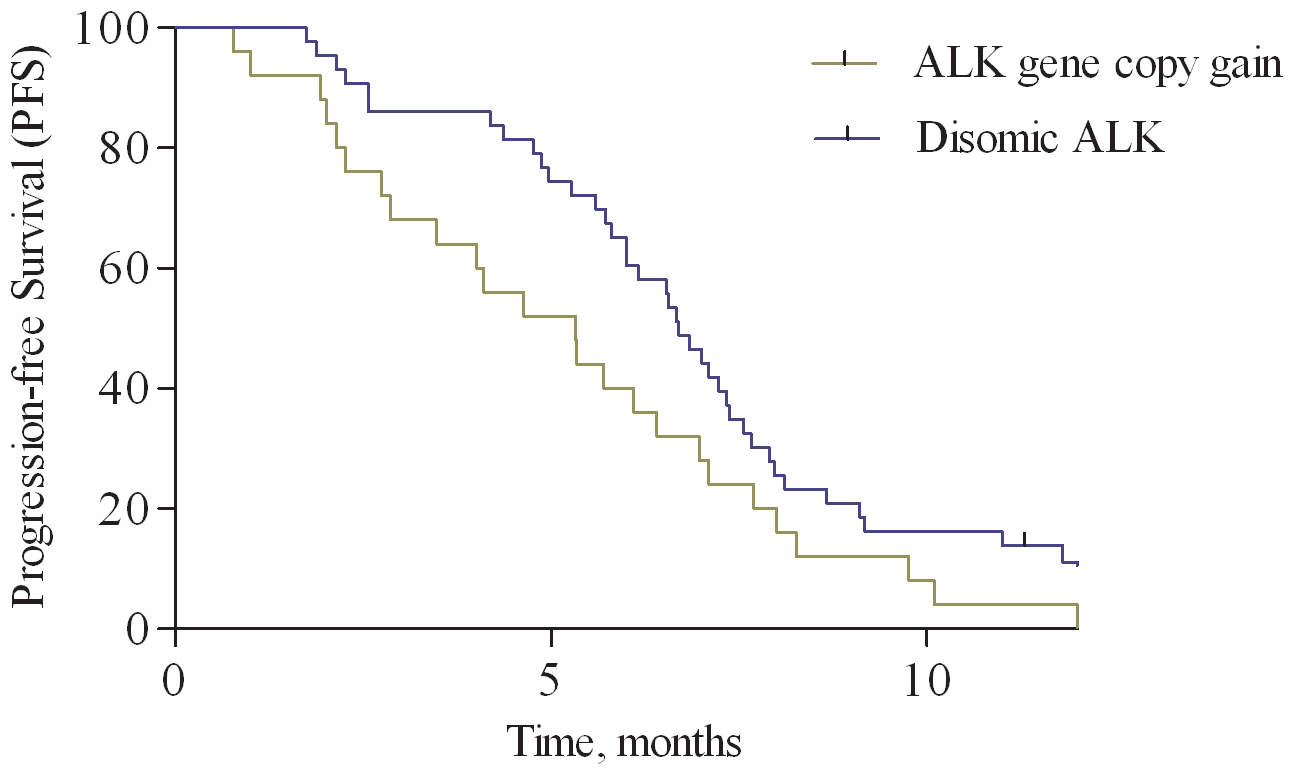
Progression-free survival analysis. Kaplan-Meier curves for progression-free survival according to ALK status: increase of gene copy number vs. disomic status.

**Figure 4:**
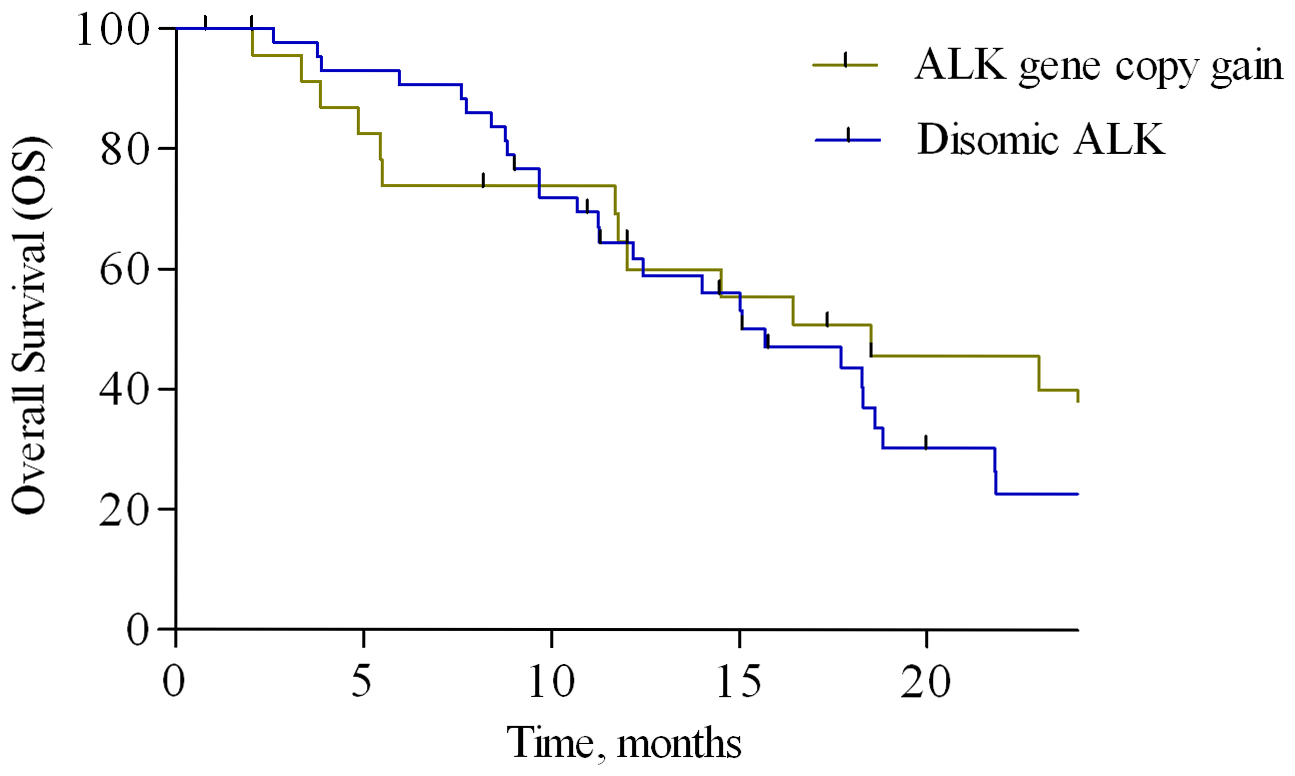
Overall survival analysis. Kaplan-Meier curves for overall survival according to ALK status: increase of gene copy number vs. disomic status.

No *ALK* translocations or amplifications were detected. *ALK* gene copy number gain was found in 25 (37%) tumors, with a median number of *ALK* signals per cell with abnormal FISH results was 3.52 (range, 3.0–5.8); disomic *ALK* status was found in 43 (63%) samples, as shown in Figure 1. Regarding correlation of *ALK* status with outcomes, only 8 of 25 (32%) patients in the group with increased *ALK* gene copy number showed a partial response according to RECIST 1.1 criteria [19], while up to 30 of 43 (70%) patients with disomic *ALK* status responded. This difference was statistically significant (p = 0.0048). PFS was significantly worsened in presence of increased *ALK* gene copy number vs. disomic *ALK* status (5.3 vs. 6.7 months; Hazard Ratio [HR] = 1.759, 95% CI, 1.013–3.053; p = 0.045; Figure 3). OS was slightly worsened in patients with increased *ALK* gene copy number, although this difference did not reach statistical significance (15.6 vs. 18.5 months; HR = 1.181, 95% CI, 0.623–1.738; p = 0.885; Figure 4).

## Discussion

International guidelines recommend *KRAS* mutation testing prior to prescribing anti-EGFR the monoclonal antibodies cetuximab and panitumumab for patients with advanced CRC and state that alternative therapy should be prescribed when mutations are detected [20]. In fact, *KRAS* mutations have been validated as predictive biomarkers of resistance to anti-EGFR treatment [4]–[7]. However, a significant percentage of patients with wild-type *KRAS* tumors fails to respond to treatment. Therefore, the identification of additional biomarkers to drive “negative” selection of patients with advanced CRC is an unmet clinical need. A more accurate treatment personalization may help to avoid unnecessary toxicity and sociosanitary costs for patients who will not benefit from treatment. Most biological factors analyzed in the attempt to improve patient selection in this setting focused either on the EGFR downstream signalling pathway or on the receptor itself. Recently, a broader mutation testing of *RAS* gene (at exons 2, 3, or 4 of both *KRAS* and *NRAS*) has been validated for treatment personalization in advanced colorectal cancer through the support of several studies [21]. Similar to RAS genes, *BRAF* also encodes proteins that act in the RAS-RAF-MAPK signalling pathway and may be involved in resistance to anti-EGFR treatment. However, these initial observations were often conflicting and limited to a small proportion of patients. For example, preliminary data on the role of BRAF status seemed promising for a straightforward application in clinical practice [22], but a large subsequent analysis from the CRYSTAL (“Cetuximab Combined With Irinotecan in First-line Therapy for Metastatic Colorectal Cancer”) fist-line trial demonstrated that BRAF mutation is a poor prognostic factor, but not a predictive one [23]. It was also demonstrated that, similarly to what observed for the oncogenic activation of the MAPK pathway, the constitutive deregulation of the *PI3KCA* could bypass the EGFR signaling pathway and be responsible of clinical resistance [13]. A previous systematic review found that *PI3KCA* exon 20 mutations was associated with a lower response rate, shorter progression-free survival and overall survival and thus may be a potential biomarker for resistance to anti-EGFR monoclonal antibodies in *KRAS* wild-type metastatic CRC, whereas *PI3KCA* exon 9 mutations seemed to have no such role [24].

Since the response rate to cetuximab is 41,2% in patients with “quadruple wild-type” tumors [7], it is evident that other biomarkers must be involved in treatment primary resistance. Alterations of membrane tyrosine kinase receptors “competing” with EGFR – such as HER-3, MET or IGFR - might play a role in treatment refractoriness, due to cross-talk in signalling downstream [25]–[27]. Despite the relevant number of preclinical observations, few clinical studies have been conducted to explore the putative function of growth factor receptor interdependence and complementarity in influencing clinical outcome with anti-EGFR treatment. Moreover, results in this research field often have been contradictory and not easily transferable to clinical practice. In fact, previous studies in this setting may have significant biases due to analysis of series with retrospectively-identified subgroups and inclusion of RAS, BRAF and PI3KCA mutant tumors. This may significantly affect the results, as the small sample size could have been clearly statistically inadequate for multiple comparisons of concomitant factors.

Recently, ALK translocation have been reported in about 2.5% of CRC characterized by C2orf44-ALK and EML4-ALK gene fusions [28], [29]. However, other ALK fusion partners have been described in non-small cell lung cancer and other tumor types, limiting the possibility to found all ALK translocations that may be present in CRC specimens. Moreover, signal enumeration in solid tumour sections by FISH is challenging to interpret and guidelines for analytical methods and scoring systems are not available for CRC, partly explaining why *ALK* gene copy number as biomarker has not been extensively investigated yet. Regarding the role of ALK in the development and progression of CRC, a recent study by Aisner et al. found marked intratumoral heterogeneity for both KRAS mutation and ALK rearrangement in CRC and in region of high-grade dysplasia [30]. Authors suggest as this evidence may create the basis for several hypotheses explaining mechanisms by which combinations of KRAS and ALK status might exist through clonal cancer evolution [30]. Regarding the prognostic role of ALK, the association between copy number alterations and clinical outcome was not extensively studied in CRC. Recently, the increase of ALK gene copy number was recognized as an independent poor prognostic factor in a retrospective series of 770 patients with CRC [31]. ALK gene copy number (amplification/gain) was found out only in 3.4% of all CRC samples studied, possibly reflecting the relatively low number of stage IV patients included and due to the statistically significant association of ALK copy number alterations with more advanced disease stage [31].

To our knowledge, our study is the first to investigate the role of ALK as a prognostic factor in patients with advanced CRC receiving cetuximab or panitumumab. In our analysis, the subgroup of patients with chemorefractory CRC and increased ALK gene copy number had ha significantly lower likelihood to respond to anti-EGFR treatment, despite a RAS-RAF-PI3KCA wild-type status. ALK status seemed to influence only the response rate and PFS, but not OS duration, thus limiting the rationale for its use as a prognostic factor. However, it must be pointed out that potential differences of OS according to ALK status may have been confounded by post-progression treatments usually prescribed at our tertiary cancer center – including chemotherapy rechallenge, regorafenib, anti-EGFR rechallenge, temozolomide in MGMT methylated tumors and molecular profiling for inclusion in phase I trials with targeted agents [32]–[34]. Furthermore, the small sample size and the lack of a control group of our study leave open the possibility that ALK copy number alterations may be a prognostic factor rather than a predictive one, since there is no way to dissect the predictive from prognostic significance. In fact, the possible predictive role of ALK gene status as key pathway of resistance to anti-EGFR treatment needs to be further confirmed through adequately powered, randomized studies. However, despite some intrinsic limitations, patients included in this analysis were obtained from a prospective database and were treated homogeneously with anti-EGFR monoclonal antibodies for chemorefractory disease. All patients were selected through a “molecular enrichment” process – and those with possible confounding RAS, BRAF, and/or PI3KCA mutations were considered ineligible.

Finally, whether high *ALK* gene copy number may represent a true predictive factor of response to ALK inhibitors was not studied in CRC. Interestingly, ALK may be a possible molecular target as part of a treatment protocol focused on control of either EGFR and ALK receptors, or the PI3KCA pathway. The possibility of using ALK inhibitors in biologically selected anti–EGFR-resistant tumors promises to be a crucial challenge for the future development of targeted therapy in CRC patients. The lack of ALK translocated cases, together with the low percentage of cells with amplification in all cases, suggests that gain of gene copy number might not be a biologically relevant event or predict response to ALK targeting molecules. Furthermore, *ALK* gene copy gain may be associated with copy number aberration of other competing genes, such as MET or EGFR itself [35], [36]. Nevertheless, these observations do not definitely rule out the potential benefit of ALK inhibitors in this population, as demonstrated in colorectal patients without EGFR protein expression that do respond to therapeutic monoclonal antibodies targeting EGFR [37].
